# The effectiveness of nonsteroidal anti-inflammatory drugs and acetaminophen in reduce the risk of amyotrophic lateral sclerosis? A meta-analysis

**DOI:** 10.1038/s41598-020-71813-1

**Published:** 2020-09-08

**Authors:** Min Cheol Chang, Sang Gyu Kwak, Jin-Sung Park, Donghwi Park

**Affiliations:** 1 Department of Rehabilitation Medicine, College of Medicine, Yeungnam University, Daegu, Republic of Korea; 2 Department of Medical Statistics, College of Medicine, Catholic University of Daegu, Daegu, Republic of Korea; 3 Department of Neurology, School of Medicine, Kyungpook National University, Kyungpook National University Chilgok Hospital, Daegu, Republic of Korea; 4 Department of Physical Medicine and Rehabilitation, Ulsan University Hospital, University of Ulsan College of Medicine, 877, Bangeojinsunghwndo-ro, Dong-gu, Ulsan, 44033 Republic of Korea

**Keywords:** Neurodegenerative diseases, Motor neuron disease

## Abstract

To test the hypothesis that aspirin, non-aspirin nonsteroidal anti-infammatory drugs (NA-NSAIDs), or acetaminophen can reduce the risk of ALS, we conducted a systematic review and meta-analysis of related previous studies. A comprehensive search was conducted on the PubMed, Embase, Cochrane Library and SCOPUS databases. It included studies published up to 29 February 2020 that fulfilled our inclusion criteria. Aspirin, acetaminophen and NA-NSAIDs use information, between the ALS and control groups, was collected for the meta-analysis. Rates of aspirin, NA-NSAID, and acetaminophen use in ALS group, compared with control group were investigated. In the results, only three studies that relate the risk of ALS to aspirin, NA-NSAIDs and acetaminophen use satisfied the inclusion criteria for the meta-analysis. Regarding aspirin, the studies did not show any statistically significant difference in aspirin use between the ALS and control groups (Odds ratio, 1.04 [95% confidence interval, 0.90–1.21]). NA-NSAIDs and acetaminophen use, however, did show up statistically significant differences in between the ALS and control groups. (Odds ratio, 0.82 [95% confidence interval, 0.73–0.91]) and (Odds ratio, 0.80 [95% confidence interval, 0.69–0.93]). However, our study has some limitations. Firstly, we only included a small number of studies. Secondly, the included studies did not control for past medical history, which may have confounded their results, and in turn, could have caused bias in our study. Thirdly, in this meta-analysis, the ALS patients were not subdivided into sporadic or familial type. Lastly, the studies also did not consider the types of NSAIDs and dosages used of each drug. For more convincing evidence regarding the effectiveness of aspirin, NA-NSAIDs and acetaminophen to reduce the risk of ALS occurrence, more qualified prospective studies are required. In conclusion, the use of NA-NSAIDs and acetaminophen is associated with a decreased risk for the development of ALS. In contrast, aspirin did not have any effect on the reduction of the risk of ALS occurrence.

## Introduction

Amyotrophic lateral sclerosis (ALS) is a progressive neurodegenerative disorder that affects the motor neurons in the spinal cord, brainstem and cerebral cortex, and which eventually leads to fatality^1,2^. Patients diagnosed with ALS show gradual-onset, progressive muscle weakness and atrophy^3^. As ALS progresses the respiratory muscles are eventually involved and this leads to respiratory failure and death^4,5^. Although the course of progression can vary, the prognosis for most patients with ALS is extremely poor. The median survival time from onset of clinical signs to death is only 20 to 48 months^6^. A small percentage, about 10% of patients, diagnosed with ALS survive for more than 10 years after the disease onset^7^. Bulbar onset and advanced age specifically are reported to have a very poor prognosis^8^. Researchers have attempted to develop disease-modifying medications, but to date the results have not been favorable. Only a few medications, such as riluzole and edaravone, are known to have the potential to improve the patient’s quality of life, as well as the survival rate for patients living with ALS^9–12^.

Evidence of inflammatory processes in the motor neurons and glial cells, resulting in neurotoxicity and associated with the development of ALS has been found in several previous studies^13–15^. Cyclooxygenase (COX) is an enzyme that is responsible for the activation of inflammation by inducing factors such as prostaglandins, thromboxane and cytokines^16–20^. Therefore, it was proposed that non-steroidal anti-inflammatory drugs (NSAIDs), which inhibit the action of COX and are typically used for reducing pain and fever, might reduce the occurrence rate of ALS and delay the progression of symptoms of ALS^21–24^. Furthermore, some studies have demonstrated that acetaminophen use also confers a neuroprotective effect^25,26^. So far, three previous studies have evaluated the effect of aspirin, non-aspirin nonsteroidal anti-inflammatory drugs (NA-NSAIDs) and acetaminophen use to the risk of developing ALS, but their results were inconsistent^21–23^.

Here, we conducted a meta-analysis of these previous studies to further explore whether aspirin, NA-NSAIDs and acetaminophen can reduce the risk of ALS development.

## Methods

### Search strategy

This meta-analysis was conducted according to the Preferred Reporting Items for Systematic Reviews and Meta-Analysis guidelines. We systematically searched the relevant literature in PubMed, Embase, Cochrane Library, and SCOPUS for studies published up to 29 February 2020. The following keywords were used in the database search: (*amyotrophic lateral sclerosis* or *ALS* or *motor neuron disease* or *MND*) and (*nonsteroidal anti-inflammatory drugs* or *NSAIDs* or aspirin or acetaminophen). The filters were used to select studies with human participants. We only included articles published in English.

### Study selection

Population-based, case–control studies that included incidence of ALS in NSAIDs and non-NSAIDs-treated populations, aspirin and non-aspirin-treated populations, and acet-aminophen and non-acetaminophen-treated populations were considered suitable for inclusion. We excluded the following: any studies with populations that did not include patients with ALS; those in which NSAIDs, aspirin or acetaminophen were not part of the intervention; those in which the measure of incidence rate in case-controlled trials was unreported; and trials in which the studied populations coexist with diseases other than ALS. We also excluded review articles, letters and case reports.

### Data extraction

All the data was independently investigated by two researchers (D.P and M.C.C) using a standardized data collection form. Discrepancies were resolved through discussions with a third investigator (S.G.K), and by referring to the original articles. In the three included studies^21–23^, data on a total of 794,622 participants were extracted (1,548 patients with ALS and 793,114 controls). Additionally, research characteristics, study design, number of study and control groups, number of included participants, diagnostic criteria, number of participants using aspirin, NA-NSAIDs, or acetaminophen in the ALS and control groups, incidence rate of aspirin, NA-NSAIDs, or acetaminophen use between the ALS and control groups, and sex ratio in the study group were investigated.

### Quality assessment

Quality assessment was performed using the Newcastle–Ottawa Scale (NOS)^27^. This scale criticizes case–control and cohort studies according to three domains, including the selection of study groups (4 items), comparability of study groups (2 items) and exposure and outcome measurement (3 items). Those with an NOS score of at least 6 were considered high-quality studies.

### Statistical analysis

The RevMan 5.3 software program (https://tech.cochrane.org/revman) was used for statistical analysis of the pooled data. In each analysis, a heterogeneity test was performed using I^2^ statistics, which measures the extent of inconsistency among the results. If p-values < 0.05, it was considered as having substantial heterogeneity, and the random-effects model was used in the analysis of the data. In contrast, if p-values were ≥ 0.05, pooled data were considered homogenous, and the fixed effects model was applied. We analyzed the odds ratio (OR) for association measures in case–control studies. The fixed and random effects models were selected according to the different heterogeneity levels of the rate ratio outcomes. Furthermore, a 95% confidence interval (CI) was used in the analysis. A p-value < 0.05 was considered statistically significant.

## Results

**Figure 1:**
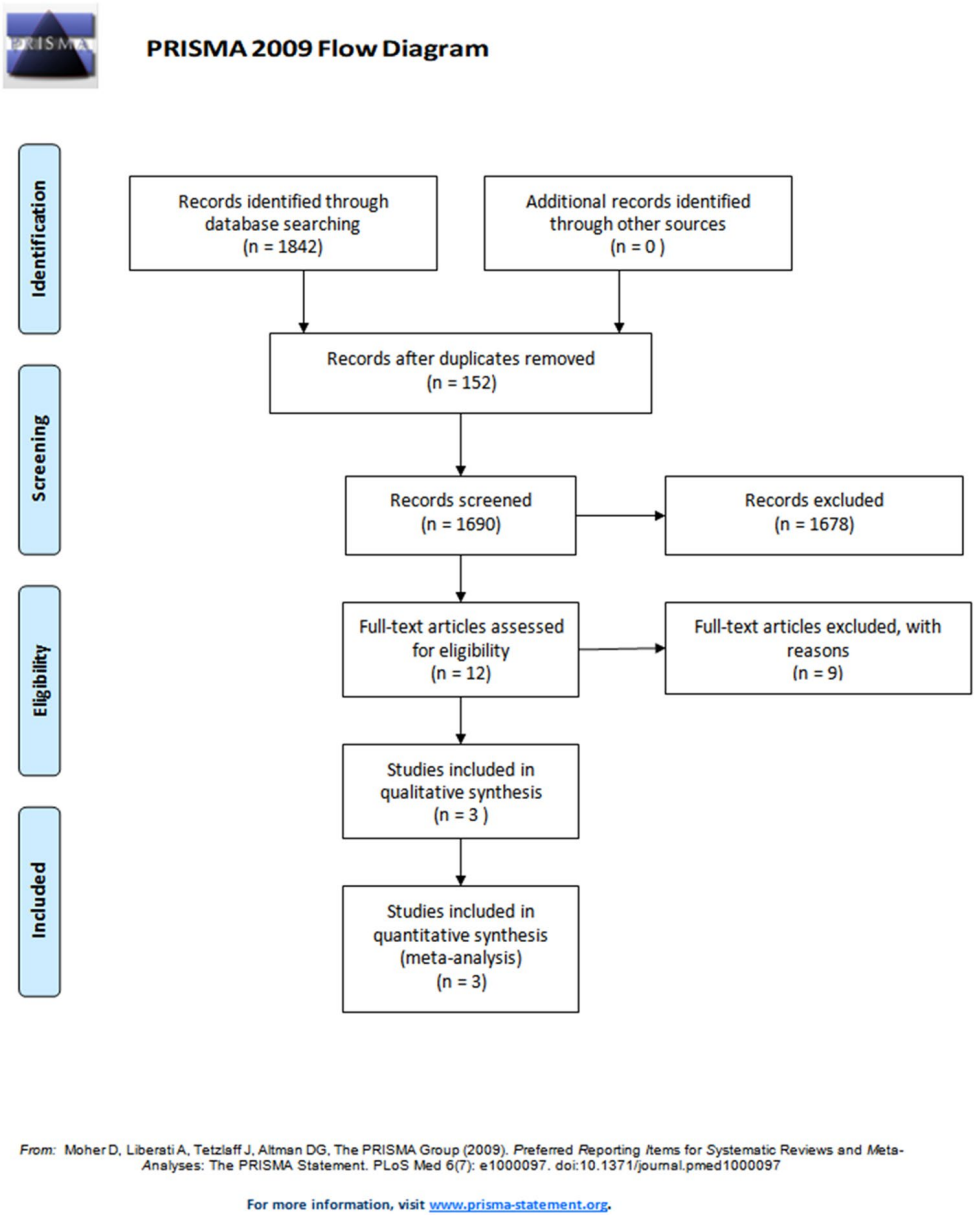
Flowchart showing the search results of the meta-analysis.

In the pooled databases, 1,842 articles were searched and 152 duplicated articles were removed (Fig. 1). After screening for eligibility, based on a review of the title and abstract, 12 articles were identified for full-text reading. After a detailed assessment 9 articles were excluded; eight studies reported insufficient results and one study was a poster presentation. Accordingly, three studies were left and finally included in our meta-analysis (Table 1). Therefore, a total of three studies on the incidence of ALS in NA-NSAIDs- and non-NA-NSAIDs-treated, aspirin- and non-aspirin-treated individuals were included to determine whether aspirin or NA-NSAIDs, or acetaminophen use affect ALS incidence. In addition, a total of two studies on the incidence of ALS in acetaminophen- and non-acetaminophen-treated individuals were included to determine whether acetaminophen use affect ALS incidence. The characteristics of the included studies are also presented in Table 1.

**Table 1:** Characteristics of case control trials assessing non-aspirin NSAIDs use and incidence of ALS in the systematic review.

Trial	Location	Participants (cases/controls)	Period of recruitment	Incidence rate (%)		Age [mean (SD)]	% males
				Cases	Controls		
Popat et al.^22^	USA	111/258	1996–2000	35.1 (39/111)	34.4 (89/258)	62.6 (12.7)	61.1
Fondell et al.^21^	USA	708/785,566	1976–1996	30.7 (217/708)	36.7 (288,323/785,566)	60.8 (7.7)	50.8
Tsai et al.^23^	Taiwan	729/7,290	2002–2008	52.7 (384/729)	56.7 (4,134/7,290)	57.4 (13.1)	61.9

### Study characteristics

In the three studies^21–23^, 1,548 participants with ALS and 793,114 participants without ALS (as controls) were recruited. Regarding to aspirin, in a study by Tsai et al.^23^, 111 out of a total of 729 patients with ALS used aspirin. Out of a total of 7,290 patients without ALS, 1,369 patients used statins (OR 0.78; 95% CI 0.63–10.96) (Table 2). In a study by Popat et al.^22^, 41 out of a total of 111 patients with ALS used aspirin and 90 out of a total of 258 patients without ALS used aspirin (OR 1.09; 95% CI 0.69–1.74). A study by Fondell et al.^21^, had 374 in a total of 708 patients with ALS use aspirin. Of a total of 784,492 patients without ALS, 405,806 used statins (OR 1.04; 95% CI 0.90–1.21). However, data on the doses of aspirin administered to the ALS and control groups was incomplete.

**Table 2:** Characteristics of case control trials assessing aspirin use and incidence of ALS in the systematic review.

Trial	Location	Participants (cases/controls)	Period of recruitment	Incidence rate (%)		Age [mean(SD)]	% males
				Cases	Controls		
Popat et al.^22^	USA	111/258	1996–2000	36.9 (41/111)	34.9 (90/258)	62.6 (12.7)	61.1
Fondell et al.^21^	USA	708/784,492	1976–1996	52.8 (374/708)	51.7 (405,806/784,492)	60.8 (7.8)	50.4
Tsai et al.^23^	Taiwan	729/7,290	2002–2008	15.2 (111/729)	18.8 (1,369/7,290)	57.4 (13.1)	61.9

Regarding NA-NSAIDs, in a study by Popat et al.^22^, out of a total of 111 patients with ALS, 39 used NA-NSAIDs. In the total of 258 patients without ALS, 89 used NA-NSAIDs (OR 1.03; 95% CI 0.65–1.64). In a study by Fondell et al.^21^, 217 out of a total of 708 patients with ALS used NSAIDs and 288,313 out of a total of 785,566 patients without ALS used NA-NSAIDs (OR 0.76; 95% CI 0.65–0.89). In a study by Tsai et al.^23^, 384 out of a total of 729 patients with ALS used NA-NSAIDs and 4,134 out of a total of 77,290 patients without ALS used NSAIDs (OR 0.85; 95% CI 0.73–0.99). However, data on the different classes of NA-NSAIDs used by the ALS and control groups were not fully available, except in a study by Tsai et al.^23^ Additionally, data on the doses of NA-NSAIDs administered to the ALS and control groups were also not fully available.

Regarding to acetaminophen, in a study by Fondell et al.^21^, 85 out of a total of 375 patients with ALS used acetaminophen and 120,900 out of a total of 417,360 patients without ALS used acetaminophen (OR 0.72; 95% CI 0.56–0.92) (Table 3). In a study by Tsai et al.^23^, 605 out of a total of 729 patients with ALS used acetaminophen and 6,183 out of a total of 77,290 patients without ALS used acetaminophen (OR 0.87; 95% CI 0.71–1.07). Once again, data on the doses of acetaminophen administered to the ALS and control groups were not fully available.

**Table 3:** Characteristics of case control trials assessing acetaminophen use and incidence of ALS in the systematic review.

Trial	Location	Participants (cases/controls)	Period of recruitment	Incidence rate (%)		Age [mean (SD)]	% Males
				Cases	Controls		
Fondell et al.^21^	USA	375/417,360	1976–1996	22.7 (85/375)	29.0 (120,900/417,360)	59.8 (8.7)	45.5
Tsai et al.^23^	Taiwan	729/7,290	2002–2008	83.0 (605/729)	84.8 (6,183/7,290)	57.4 (13.1)	61.9

### Risk of bias

The three studies were assessed using NOS and all were rated with 8 stars, which is considered as having a relatively high quality (selection of subjects, 4 stars; comparability of groups, 2 stars; assessment of outcome, 2 stars).

### Meta-analysis results

**Figure 2:**
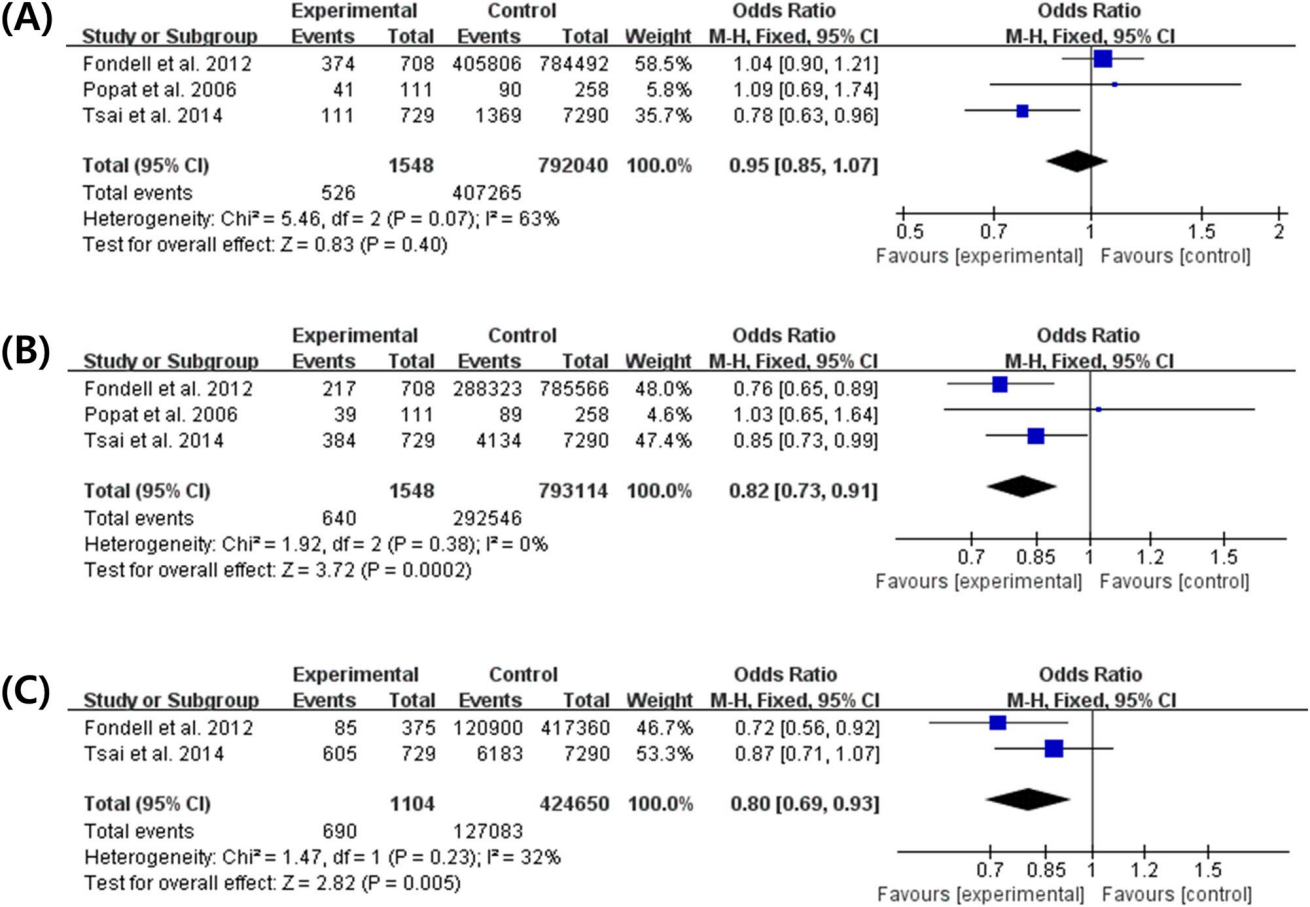
Forest plot of aspirin, non-aspirin non-steroidal anti-inflammatory drugs (NA-NSAIDs), and acetaminophen use in patients with amyotrophic lateral sclerosis versus controls. Note: Three studies were included. Regarding to aspirin, there was no statistically significant difference in aspirin use between the ALS and controls groups (Odds ratio, 1.04 [95% confidence interval, 0.90–1.21]) (**A**). Regarding to non-aspirin NSAIDs and acetaminophen, however, there were statistically significant differences in NA-NSAIDs (**B**) and acetaminophen (**C**) use between the ALS and controls groups. (Odds ratio, 0.82 [95% confidence interval, 0.73–0.91]) vs. (Odds ratio, 0.80 [95% confidence interval, 0.69–0.93]).

In the meta-analysis of aspirin use, a fixed effects model was adopted. The OR of the three studies is presented in Fig. 2A. There was no statistical difference in ALS incidence between the aspirin and non-aspirin groups (OR 0.94 [95% CI 0.75–1.18]). In the meta-analysis of NA-NSAIDs, a fixed effects model was adopted, because the P-values for heterogeneity of all our analyses were ≥ 0.05. The OR of the three studies is presented in Fig. 2B. There was a statistically significant difference in ALS incidence between the NA-NSAIDs and non-NA-NSAIDs groups (OR 0.82 [95% CI 0.73–0.91]). In the meta-analysis of acetaminophen, a fixed effects model was adopted, because the P-values for heterogeneity of all our analyses were ≥ 0.05. The OR of the three studies is presented in Fig. 2C. There was a statistically significant difference in ALS incidence between the acetaminophen and non-acetaminophen groups (OR 0.80 [95% CI 0.69–0.93]).

### Publication bias

**Figure 3:**
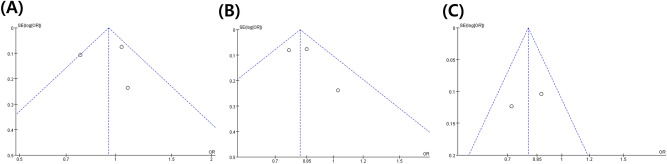
Graphic funnel plot of included studies. Aspirin (**A**), NA-NSAIDs (**B**), and acetaminophen (**C**).

Based on a few distinct methods, two of the authors (DP and MCC) individually assessed the risk of bias. We determined the risk of publication bias using a funnel plot and Egger’s test^28^. The Egger`s test of aspirin and NA-NSAIDs were not significant (p = 0.4596, p = 0.9673). Moreover, funnel plots of aspirin, NA-NSAIDs, and acetaminophen meta-analysis were produced to investigate the risk of publication bias, as shown in Fig. 3. Performing a visual inspection suggested funnel plot symmetry, suggesting there is no risk of publication bias^28^.

## Discussion

In our meta-analysis review we evaluated the association between the use of aspirin, NA-NSAIDs and acetaminophen and the occurrence of ALS. We found that NA-NSAIDs and acetaminophen significantly reduced the occurrence of ALS. The effect sizes were 0.82 and 0.80 for NA-NSAIDs and acetaminophen, respectively. Based on Cohen’s study^29^, these effect sizes indicate that NA-NSAIDs and acetaminophen have very positive effects for reducing the risk of ALS development.

COX plays a pivotal role in the neurotoxicity seen in ALS^30,31^, and it includes two isoforms: COX-1 and COX-2^16,17^. While COX-1 is constitutively expressed in most tissues and produces prostanoids which sustain homeostasis in several organs and cells, i.e. the gastrointestinal tract, kidneys, and platelets^18–20^; COX-2 on the other hand is responsible for the transcriptional control of pro- and anti-inflammatory cytokines^18–20^. COX-2 activates the synthesis of prostaglandin and thromboxane, which in turn stimulates the secretion of inflammatory cytokines, such as tumor necrosis factor-alpha and interleukin-1, and induces the inflammatory pathway cytokines^18–20^. Non-aspirin NSAIDs inhibit the activity of COX-2, consequently inhibiting the inflammatory process by inhibiting prostaglandin synthesis^30,32–34^. This explains why non-aspirin NSAIDs would confer neuroprotective effects which seem to suppress the development of ALS.

Acetaminophen only confers weak effects on the inhibition of the action of COX-2 in humans^25^. Due to its minor anti-inflammatory effect, acetaminophen is not classified as an NSAID^25^. In a previous study, however, acetaminophen reportedly has the potential of exerting anti-oxidant effects to protect neurons from oxidative stress, i.e. by reducing the release of cytokines and chemokines such as tumor necrosis factor alpha, interleukin-1, and macrophage inflammatory protein, from the neurons by a superoxide releasing oxidant stressor^26^. In that study, acetaminophen was shown to have pro-survival effects on neurons by increasing expression of the anti-apoptotic protein Bcl2 in brain neurons and by decreasing the superoxide-induced elevation of the pro-apoptotic protein, cleaved caspase 3^26^. Considering that the ROS-related genes, such as SOD1 (copper zinc superoxide dismutase 1^35^, are important genetic causes of ALS, the anti-oxidant effect of acetaminophen seems to be an important mechanism in suppressing the development and progression of ALS.

In contrast to NA-NSAIDs and acetaminophen, in our meta-analysis, aspirin was not found to have an effect on the development of ALS. Aspirin, or acetylsalicylic acid, belongs to NSAID family and inhibits the actions of both COX-1 and COX-2 irreversibly^36,37^. However, while high doses of aspirin (≥ 1,000 mg per day) inhibits the COX-2 isozyme more strongly than COX-1; low doses of aspirin (≤ 100 mg per day) inhibits COX-1 more than it inhibits COX-2^38,39^. Low-dose aspirin is used to prevent coronary syndromes and cerebral infarcts in patients who are at high risk of developing these disorders^38,40^. While high-dose aspirin use does confer anti-inflammatory effects, it is rarely used in clinical practice due to the high risk of adverse effects, which include gastric ulcers and hemorrhagic stroke^39,41^. Although the doses in the three included reports in our meta-analysis were not described, we suspect that low-dose aspirin was used by the subjects of these studies. Therefore, potential neuroprotective effects by blocking the action of COX-2 seem to have been insignificant.

In conclusion, we found that the use of NA-NSAIDs and acetaminophen is associated with a decreased risk of development of ALS, and these medications seem to confer neuroprotective effects. In contrast, aspirin did not have any effect on the reduction of the risk of ALS occurrence. Our study is the first meta-analysis to evaluate the association between aspirin, NA-NSAID, and acetaminophen use and the risk of ALS. However, our study has some limitations. Firstly, we only included a small number of studies. Secondly, the included studies did not control for past medical history, which may have confounded their results, and in turn, could have caused bias in our study. Therefore, to confirm it, further studies using Mendelian randomization analysis may be necessary. Thirdly, in this meta-analysis, the ALS patients were not subdivided into sporadic or familial type. Lastly, the studies also did not consider the types of NSAIDs and dosages used of each drug. For more convincing evidence regarding the effectiveness of aspirin, NA-NSAIDs and acetaminophen to reduce the risk of ALS occurrence, more qualified prospective studies are required.

## Data Availability

Data availability depends on agreement from each of the participating studies, subject to their regulatory requirements and appropriate data sharing arrangements.

## References

[1] Kiernan MC (2011). Amyotrophic lateral sclerosis. Lancet.

[2] Renton AE, Chio A, Traynor BJ (2014). State of play in amyotrophic lateral sclerosis genetics. Nat. Neurosci..

[3] Visser J (2007). Disease course and prognostic factors of progressive muscular atrophy. Arch. Neurol..

[4] Niedermeyer S, Murn M, Choi PJ (2019). Respiratory failure in amyotrophic lateral sclerosis. Chest.

[5] Park JS, Park D (2017). The terminal latency of the phrenic nerve correlates with respiratory symptoms in amyotrophic lateral sclerosis. Clin. Neurophysiol..

[6] Paulukonis ST (2015). Survival and cause of death among a cohort of confirmed amyotrophic lateral sclerosis cases. PLoS ONE.

[7] Oliveira AS, Pereira RD (2009). Amyotrophic lateral sclerosis (ALS): three letters that change the people's life. Forever. Arq. Neuropsiquiatr..

[8] Knibb JA (2016). A clinical tool for predicting survival in ALS. J. Neurol. Neurosurg. Psychiatry.

[9] Heiman-Patterson TD, Miller RG (2006). NIPPV: a treatment for ALS whose time has come. Neurology.

[10] Thakore NJ, Lapin BR, Pioro EP (2020). Stage-specific riluzole effect in amyotrophic lateral sclerosis: a retrospective study. Amyotrophic Lateral Sclerosis.

[11] Writing G, Edaravone ALSSG (2017). Safety and efficacy of edaravone in well defined patients with amyotrophic lateral sclerosis: a randomised, double-blind, placebo-controlled trial. Lancet Neurol..

[12] Miller RG, Mitchell JD, Moore DH (2012). Riluzole for amyotrophic lateral sclerosis (ALS)/motor neuron disease (MND). Cochrane Database Syst. Rev..

[13] Birger A (2019). Human iPSC-derived astrocytes from ALS patients with mutated C9ORF72 show increased oxidative stress and neurotoxicity. EBioMedicine.

[14] Nakaya T, Maragkakis M (2018). Amyotrophic Lateral Sclerosis associated FUS mutation shortens mitochondria and induces neurotoxicity. Sci. Rep..

[15] Shenouda M, Zhang AB, Weichert A, Robertson J (2018). Mechanisms associated with TDP-43 neurotoxicity in ALS/FTLD. Adv. Neurobiol..

[16] Kuehl FA, Egan RW (1980). Prostaglandins, arachidonic acid, and inflammation. Science.

[17] Morita I (2002). Distinct functions of COX-1 and COX-2. Prostaglandins Other Lipid Mediat..

[18] Kirkby NS (2016). Systematic study of constitutive cyclooxygenase-2 expression: role of NF-kappaB and NFAT transcriptional pathways. Proc. Natl. Acad. Sci. USA.

[19] Ricciotti E, FitzGerald GA (2011). Prostaglandins and inflammation. Arterioscler Thromb. Vasc. Biol..

[20] Zidar N (2009). Cyclooxygenase in normal human tissues: is COX-1 really a constitutive isoform, and COX-2 an inducible isoform?. J. Cell Mol. Med..

[21] Fondell E (2012). Non-steroidal anti-inflammatory drugs and amyotrophic lateral sclerosis: results from five prospective cohort studies. Amyotrophic Lateral Sclerosis.

[22] Popat RA (2007). Effect of non-steroidal anti-inflammatory medications on the risk of amyotrophic lateral sclerosis. Amyotrophic Lateral Sclerosis.

[23] Tsai CP, Lin FC, Lee JK, Lee CT (2015). Aspirin use associated with amyotrophic lateral sclerosis: a total population-based case-control study. J. Epidemiol..

[24] Barneoud P, Curet O (1999). Beneficial effects of lysine acetylsalicylate, a soluble salt of aspirin, on motor performance in a transgenic model of amyotrophic lateral sclerosis. Exp. Neurol..

[25] Botting RM (2000). Mechanism of action of acetaminophen: is there a cyclooxygenase 3?. Clin. Infect. Dis..

[26] Tripathy D, Grammas P (2009). Acetaminophen inhibits neuronal inflammation and protects neurons from oxidative stress. J. Neuroinflamm..

[27] Stang A (2010). Critical evaluation of the Newcastle-Ottawa scale for the assessment of the quality of nonrandomized studies in meta-analyses. Eur. J. Epidemiol..

[28] Duval S, Tweedie R (2000). Trim and fill: a simple funnel-plot-based method of testing and adjusting for publication bias in meta-analysis. Biometrics.

[29] Faul F, Erdfelder E, Lang AG, Buchner A (2007). G*Power 3: a flexible statistical power analysis program for the social, behavioral, and biomedical sciences. Behav. Res. Methods.

[30] Seibert K (1995). Mediation of inflammation by cyclooxygenase-2. Agents Actions Suppl..

[31] Xia Q (2015). Induction of COX-2-PGE2 synthesis by activation of the MAPK/ERK pathway contributes to neuronal death triggered by TDP-43-depleted microglia. Cell Death Dis..

[32] Carty ML (2011). Ibuprofen inhibits neuroinflammation and attenuates white matter damage following hypoxia-ischemia in the immature rodent brain. Brain Res..

[33] Klegeris A, McGeer PL (2002). Cyclooxygenase and 5-lipoxygenase inhibitors protect against mononuclear phagocyte neurotoxicity. Neurobiol. Aging.

[34] Pompl PN (2003). A therapeutic role for cyclooxygenase-2 inhibitors in a transgenic mouse model of amyotrophic lateral sclerosis. FASEB J.

[35] Carrera-Julia S, Moreno ML, Barrios C, de la Rubia Orti JE, Drehmer E (2020). Antioxidant alternatives in the treatment of amyotrophic lateral sclerosis: a comprehensive review. Front. Physiol..

[36] Kerola M (2009). Effects of nimesulide, acetylsalicylic acid, ibuprofen and nabumetone on cyclooxygenase-1- and cyclooxygenase-2-mediated prostanoid production in healthy volunteers ex vivo. Basic Clin. Pharmacol. Toxicol..

[37] Baigent C, Patrono C (2003). Selective cyclooxygenase 2 inhibitors, aspirin, and cardiovascular disease: a reappraisal. Arthritis Rheum..

[38] Warner TD, Nylander S, Whatling C (2011). Anti-platelet therapy: cyclo-oxygenase inhibition and the use of aspirin with particular regard to dual anti-platelet therapy. Br. J. Clin. Pharmacol..

[39] Xie Y (2019). Dose-dependent roles of aspirin and other non-steroidal anti-inflammatory drugs in abnormal bone remodeling and skeletal regeneration. Cell Biosci..

[40] Seidu S (2019). Aspirin has potential benefits for primary prevention of cardiovascular outcomes in diabetes: updated literature-based and individual participant data meta-analyses of randomized controlled trials. Cardiovasc. Diabetol..

[41] Flower RJ (1974). Drugs which inhibit prostaglandin biosynthesis. Pharmacol. Rev..

